# Using the modified socio-ecological model to address stigma against non-suicidal self-injury among adolescents in Malaysia

**DOI:** 10.3389/fpsyt.2023.1204704

**Published:** 2023-06-15

**Authors:** Chern Choong Thum, Rahima Dahlan, Yen Jun Wong

**Affiliations:** ^1^Hospital Sultan Abdul Aziz Shah, Universiti Putra Malaysia, Serdang, Selangor, Malaysia; ^2^Faculty of Medicine and Health Sciences, Universiti Putra Malaysia, Serdang, Malaysia; ^3^School of Pharmacy, Monash University Malaysia, Subang Jaya, Selangor, Malaysia

**Keywords:** socio-ecological system, non-suicidal self-injury, NSSI, NSSI-related stigma, adolescents

## Introduction

Non-suicidal self-injury is defined as an intentional injury to one's body without a desire to cause death (1). It may involve acts that include, but are not limited to, damaging one's own skin and self-poisoning for purposes that are not socially sanctioned (2). In this article, the term “non-suicidal self-injury” (NSSI) and its acronym NSSI are preferred because it is a diagnostic entity in the fifth edition of the Diagnostic and Statistical Manual of Mental Disorders (DSM-5) (3). NSSI usually follows a behavioral cycle. It often begins with negative emotions such as depression, anxiety, or stress. These emotions can trigger the urge to engage in NSSI, as individuals use self-harm as a coping mechanism to regulate their emotions (4). This can then trigger a new cycle of negative emotions, vulnerability factors, urges, and self-injury. Breaking the cycle of NSSI can be challenging and often requires the support of mental health professionals and the development of healthy coping mechanisms to regulate negative emotions (5).

### NSSI epidemiology

While the lifetime prevalence of NSSI varies widely, it is highest in adolescents (6–8). This is thought to be due to biological changes during puberty, which is confirmed by the typical onset of NSSI at 12–14 years of age (9). Reports of NSSI have been found to decrease as adolescents get older, suggesting that adolescence is the most important period for intervention (10). Franzen et al. report that NSSI accounts for most emergency admissions to child and adolescent psychiatry (11).

There are reports that present NSSI as a coping strategy to manage emotional dysregulation (12, 13). The causes of NSSI are multifactorial and include neurobiological, psychological, and environmental factors, while risk factors for NSSI in adolescents include low health literacy, adverse childhood experiences, and bullying (14, 15). Oliveira Costa and colleagues postulated that NSSI is used as a coping strategy for emotional dysregulation that provides immediate relief, which can lead to a repetitive pattern of self-injurious behavior (16). In addition, the prevalence of NSSI among adolescents was reported to have increased after the COVID-19 pandemic (17). This is significant because it was reported that the increase in NSSI repetitions would lead to higher risk behaviors such as suicide (18).

In 2011, the prevalence of NSSI among Malaysian children and adolescents was 12 per 100,000 population (19). While there are no data on NSSI prevalence specifically among adolescents in Malaysia, the 2017 National Health and Morbidity Survey shows that suicidal ideation among 13- to 17-year-olds increased from 7.9% in 2012 to 10% in 2017 (20). Similarly, there was an increase in suicide plans and attempts in the surveyed population, with suicide plans increasing from 6.4% in 2012 to 7.3% in 2017 in addition to suicide attempts increasing from 6.8% in 2012 to 6.9% in 2017.

In a study conducted among Malaysian teenagers aged 13 to 17, Mohammad and colleagues found that the prevalence of NSSI varied from 11.9 percent to 16.3 percent. Over 15% of respondents, on average, had undergone NSSI. NSSI behavior was present in somewhat more girls (16.1%) than males (14.9%) (14.2 percent). NSSI was claimed to have affected 16.2% of respondents of Indian ethnicity, 15% of respondents of Malay ethnicity, 11.6% of respondents of Chinese ethnicity, and 28.9% of respondents of other ethnic groups (21).

Studies on 13- to 16-year-old students of Chinese-Malaysian ethnicity identified prevalence between 56.8–68.0%. Female students reported to have higher rates, and the most frequently reported NSSI behaviors to be pulling one's own hair out and using erasers to erase one's own skin and hitting oneself on purpose (22). A study by Ganaprakasam and colleagues revealed 20.6% of 14- to 17-year-old Malaysian students engaged in NSSI behavior and highlighted psychological distress to be a predictor of NSSI among adolescents (23).

Remarkable progress has been made in decriminalizing suicide in Malaysia. Previously, attempted suicide was considered a criminal offense under Section 309 of the Malaysian Penal Code, which was a barrier for people in need of psychosocial support (24). However, in recent years, efforts to decriminalize have increased, driven by mental health experts and organizations calling for suicide to be treated as a public health issue rather than a criminal offense. In April 2023, a major legislative milestone was reached when a proposal to decriminalize suicide was introduced in the Malaysian Parliament. This marked a critical shift in Malaysia's approach to mental health and underscored the importance of compassionate solutions over punitive measures (25).

### Association of NSSI with stigma

Another major challenge associated with NSSI is the multidimensional social stigma that can limit the ability and access of individuals affected by NSSI to necessary medical and psychological treatment (26). A study has demonstrated that people without a history of NSSI demonstrate a strong and significant implicit and explicit negative bias toward individuals who displayed NSSI behaviorism (27). This multidimensional nature of NSSI means interventions should be multifaceted and take into consideration the various factors that contribute to this behavior. This includes addressing the stigma associated with NSSI, which can limit access to needed medical and psychological treatment.

NSSI, like many other mental illnesses, often faces stigma in various societies and cultures, including Malaysia. This stigma often results from a complex interaction of factors such as lack of understanding, societal norms, and cultural or religious beliefs. In the Malaysian context, individuals who participate in NSSI often face both external and internal forms of stigma (28). External stigma refers to societal attitudes that perpetuate stereotypes and discrimination, while internal or self-stigma can lead to feelings of shame and self-hatred (12). Thus, these individuals not only face societal misperceptions and prejudice, but also struggle with their own internalized stigma, which exacerbates their emotional distress.

It is useful to consider both the legal and religious perspectives when considering NSSI stigmatization in Malaysia. Legally, the move to decriminalize suicide attempts has the potential to encourage more help-seeking behavior. Religiously, Fernandez and colleagues argue that all primary religions in Malaysia disprove self-injury and instead encourage them to seek professional help (29), while another study has shown religiosity providing a positive effect on resilience and self-efficacy (30). Further studies may be required to address this issue from the NSSI point of view. By eliminating stigma and providing appropriate treatment and support, we can help people who commit NSSI improve their mental health and quality of life.

The modified socio-ecological model developed by Chang and colleagues can be a useful framework for addressing the stigma of NSSI (31). Applying the modified socio-ecological model in Malaysia context can help address stigma at multiple levels. For example, interventions can focus on improving mental health literacy at the individual level, promoting supportive behaviors within relationships, implementing mental health programs in schools or workplaces at the community level, and advocating for policies that promote mental health at the societal level.

Efforts to address mental health stigma, such as NSSI, often include strategies such as public awareness campaigns, school-based programs, community initiatives, legislative and policy changes, professional training for health care providers, and ongoing research and evaluation. However, literature on specific measures addressing NSSI-related stigma in Malaysia is scarce, which reflects the need for relevant studies to be carried out on this topic.

### Using the modified socio-ecological model to address stigma against NSSI

**Figure 1:**
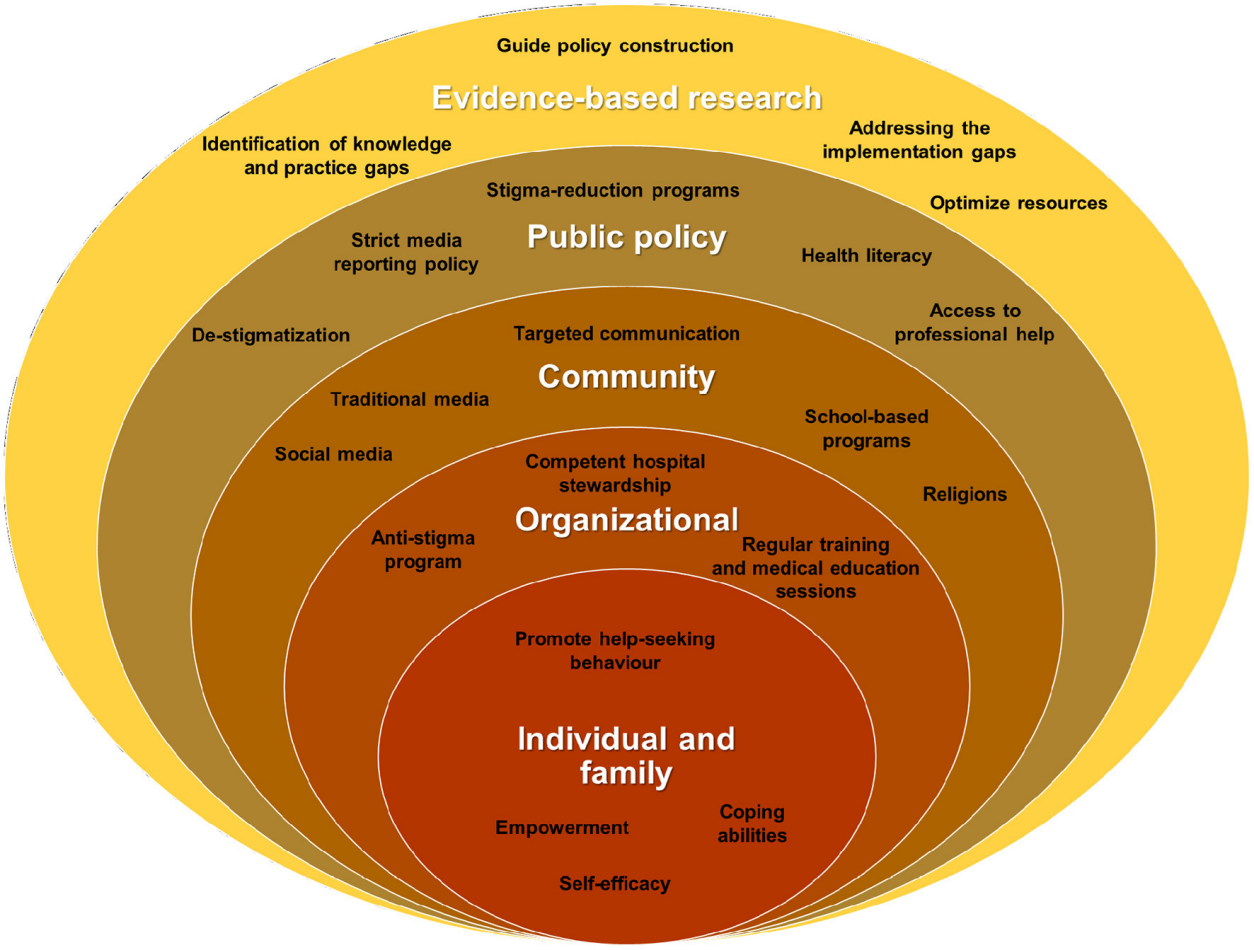
Using a modified social ecological model in addressing NSSI-related stigma.

We recommend adopting the modified socio-ecological model proposed by Chang and colleagues to better structure and illustrate anti-stigma interventions that can be implemented for self-injurious behavior (31). The model, originally proposed by Salihu et al., was developed to reduce barriers and challenges in randomized controlled trials among minority and underserved communities (32). The framework recognizes that individual behavior is influenced by multiple influencing factors, including individual, interpersonal, institutional, community, and political levels. In the modified version, both the individual and interpersonal levels are grouped under the category of “individual and family” and evidence-based research is included as a fifth level. This model suggests that effective prevention of self-injurious behavior requires consideration of the multiple levels of influence that may contribute to this behavior (Figure 1).

The socio-ecological model is well suited to the treatment of non-suicidal self-injury (NSSI) in Malaysia because it considers the cultural diversity of the country and provides a multi-faceted approach that incorporates culturally specific factors. This model provides a comprehensive understanding of the multiple factors that contribute to the stigma of NSSI and allows for the development of more holistic interventions. It helps identify specific contextual elements at different levels, from the individual to society, facilitating the development of tailored interventions that address the unique needs of different communities in Malaysia. It also highlights the role of societal factors such as policies and social norms that contribute to stigma, which in turn helps advocate for necessary policy changes to reduce stigma and improve support for people affected by NSSI.

## Discussion

### Individual and family level

Taylor et al. suggest individuals engaging in NSSI seek specific objectives, with intrapersonal reasons (such as emotional regulation) and interpersonal reasons (to communicate distress and seek support) being among the reasons (13). Tan et al. note that individuals with NSSI not only use it for emotional regulation but suffer from adverse fallout following the act. Participants reported self-hate as they needed to self-injure to manage their emotions, and experienced felt stigma (12). While family members of affected individuals may experience courtesy stigma, families and family functioning of affected individuals may too react strongly and develop negative attitudes to those who engaged in NSSI (33, 34). This model places emphasis on help-seeking behavior, both individually and with support from family members, as an initiative that may promote empowerment, self-efficacy, and coping abilities. Besides, positive family support in help-seeking behavior may improve health outcomes in the long run (35).

### Organizational level

Medical illness-related stigma has been identified to prevent patients from seeking effective healthcare (36). Mckenna reports that patients presenting to the healthcare facilities with NSSI receive higher levels of perceived stigma as compared with patients with other forms of chief complaints (37). In a study by Lloyd-Richardson, health care providers were found to view NSSI as attention-seeking behavior rather than a sign of a mental disorder (38). This could lead to dismissive or stigmatizing attitudes toward individuals who commit NSSI and limit their willingness to seek appropriate health services. This study suggests that addressing the stigma of NSSI in health care settings is important to improve access to appropriate treatment and support for individuals who engage in this behavior. As Chang et al. pointed out, competent hospital management is needed to create a compassionate and friendly environment (31). This would encourage individuals with NSSI to seek the medical and psychological help they need. To achieve this, training modules for health care workers should include an anti-stigma program, and regular continuing medical education sessions need to focus on improving knowledge and correcting poor understanding toward individuals presenting with NSSI or suicide attempts (39, 40). Health care workers should also be sensitive about the terms they use (41).

### Community level

People living in the same community are likely to share similar values, beliefs, and attitudes, both positive and negative. This includes stigma and discrimination toward a certain condition. Thus, our model calls for buy-in from the community level. When addressing the topic of stigma and NSSI, we must begin with the cultural and religious aspect.

Working with religious leaders can indeed be a strategic approach to addressing the stigma associated with NSSI. This is particularly important in societies where religion plays an important role in people's lives, as in Malaysia. Religious leaders often enjoy the respect and trust of their communities. They can use their influential position to promote understanding, compassion, and acceptance of those struggling with mental health problems and to counter stigma. They can also encourage people to seek help by providing information about available resources and emphasizing the importance of mental health alongside mental wellbeing.

Targeted communication would be a good move to dispel stigma in the community. In this context, “targeted communication” means the creation and dissemination of messages specifically designed to appeal to populations based on their unique characteristics and needs. By combining traditional and social media channels approaches, anti-stigma campaigns can ensure broad reach while providing tailored messages that can promote understanding of NSSI and reduce associated stigma (42). Considerations can also be put into school-based programs, seeing as adolescents spend the bulk of their time outside their home at school (43).

### Public policy level

Structural stigma occurs when laws, policies, and even the judicial system are not neutral in the distribution of resources. Therefore, people affected by stigma have an important role to play in ensuring that public policies are equitable.

In Malaysia, criminalization of suicide attempts is one of the barriers preventing individuals who engage in NSSI from seeking help. Although there is evidence that suicide attempts are often desperate pleas for help (24), they are still classified as a criminal act. The Malaysian government's initiative to decriminalize suicide by proposing to repeal Section 309 of the Penal Code could therefore be a constructive step to facilitate help-seeking by individuals affected by NSSI (25). Priority should be given to planning, funding, and implementing programs to reduce stigma in all segments of society (44).

The Malaysian Ministry of Health, recognizing the potential impact of media coverage of suicide, published a guide to media coverage of suicide in 2011 (45). This guideline likely included important recommendations for journalists. However, this guideline does not extend to the reporting of NSSI behaviors. NSSI, although distinct from suicide, may also be a sensitive topic that needs to be handled carefully in media reports, and strict guidelines would be helpful.

Next, focus on improving health literacy first and access to care second, so that patients know why they need help and how to access it. A national consensus on eliminating the stigma of NSSI could encourage help-seeking behaviors.

### Evidence-based research level

The incorporation of evidence-based research into any model is a necessity, as a well-structured policy backed by evidence reduces wastage and optimizes resources (46). Thus far, there has been an abundance of research published on the topic of stigma associated with NSSI, and policymakers should utilize this resource appropriately firstly identify gaps in the system and secondly use it to guide policy construction. Further resources ought be directed toward gaps identified in knowledge, so that we are able to address this issue judiciously.

## Conclusion

In summary, the stigmatization of NSSI is a significant problem that can prevent affected individuals from seeking the medical and psychological treatment they need. Addressing this problem requires a multifaceted and multidimensional approach that uses the modified socio-ecological model to understand how stigma arises at different levels of society. By implementing targeted interventions at each level of the model, we can work to eliminate the stigma of NSSI, promote greater understanding and support for people who engage in this behavior, and ensure that they have access to the medical and psychological support they need. Further research and development of effective interventions to reduce stigma is critical, as this can have a significant impact on improving the lives of individuals who commit NSSI.

## Author contributions

RD contributed to the conception and design of the manuscript. CT contributed to the conception and wrote sections of the manuscript. YW wrote sections of the manuscript. All authors contributed to manuscript revision, read, and approved the submitted version.
